# GaInP nanowire arrays for color conversion applications

**DOI:** 10.1038/s41598-020-79498-2

**Published:** 2020-12-22

**Authors:** Dennis Visser, Yohan Désières, Marcin Swillo, Eleonora De Luca, Srinivasan Anand

**Affiliations:** 1 Department of Applied Physics, KTH Royal Institute of Technology, Electrum 229, 164 40 Kista, Sweden; 2 University Grenoble Alpes, CEA, LETI, MINATEC Campus, 38054 Grenoble, France; 3 Department of Applied Physics, KTH Royal Institute of Technology, Roslagstullsbacken 21, 106 91 Stockholm, Sweden

**Keywords:** Nanowires, Optics and photonics

## Abstract

Color conversion by (tapered) nanowire arrays fabricated in GaInP with bandgap emission in the red spectral region are investigated with blue and green source light LEDs in perspective. GaInP nano- and microstructures, fabricated using top-down pattern transfer methods, are derived from epitaxial Ga_0.51_In_0.49_P/GaAs stacks with pre-determined layer thicknesses. Substrate-free GaInP micro- and nanostructures obtained by selectively etching the GaAs sacrificial layers are then embedded in a transparent film to generate stand-alone color converting films for spectrophotometry and photoluminescence experiments. Finite-difference time-domain simulations and spectrophotometry measurements are used to design and validate the GaInP structures embedded in (stand-alone) transparent films for maximum light absorption and color conversion from blue (450 nm) and green (532 nm) to red (~ 660 nm) light, respectively. It is shown that (embedded) 1 μm-high GaInP nanowire arrays can be designed to absorb ~ 100% of 450 nm and 532 nm wavelength incident light. Room-temperature photoluminescence measurements with 405 nm and 532 nm laser excitation are used for proof-of-principle demonstration of color conversion from the embedded GaInP structures. The (tapered) GaInP nanowire arrays, despite very low fill factors (~ 24%), can out-perform the micro-arrays and bulk-like slabs due to a better in- and out-coupling of source and emitted light, respectively.

## Introduction

Color conversion materials are interesting for many applications in the field of optoelectronics, e.g., solid-state lighting^1–8^, displays^2^, solar cells^9^, biomedicine^10^, and optical communication^11^. Well known is the use of phosphors for wavelength down-conversion in high-brightness blue light emitting diodes (LEDs) to obtain white light illumination (white LEDs)^3,6–8^. Absorption in phosphors like Yag:Ce is however low and thick layers, typically hundreds of microns, are needed to absorb source blue light significantly. Additionally, the use of dyes^12,13^ and wavelength conversion particles/polymers (WCP)^9,14^ are widely reported. In general, these materials absorb light at energies higher than the characteristic energy gaps and emit light at specific wavelengths (colors) due to a fluorescence/luminescence process. Pigments and dyes are sensitive to photobleaching and are prone to degradation over time. Recently, colloidal semiconductor quantum dots (Q-dots) have gained a lot of ground as color conversion materials^15–18^ due to improvements in quantum yield, color purity, and synthesis methods. The emitted wavelength can be tuned by the choice of Q-dot material and size. Already, Q-dots are successfully used for color conversion in commercial LED displays^19^. However, for micro-display applications technological improvements in Q-dot color conversion films are necessary. Currently used Q-dot films require relatively thick layers (> 5 μm) and a high density of Q-dots to absorb all the source light (e.g., blue), which is a limitation for micro-displays^19^. Q-dots need to be properly dispersed in a transparent medium to avoid optical trapping, re-absorption, and aggregation induced quenching. Recently, perovskite nanocrystals^20–24^ have emerged as promising candidate materials for color conversion applications. However, the stability of perovskites is usually an issue and needs to be improved for practical applications. Other reported color conversion approaches are based on non-linear optical processes^25–27^, which generally have low efficiencies. Photon recycling based on conventional direct bandgap semiconductors as converters in blue LEDs is an attractive alternative^28–30^ wherein the above bandgap light (blue) is absorbed to generate characteristic band-edge emission of the converter. Owing to their direct bandgaps such converters can be efficient light absorbers and emitters and require relatively less material (e.g., the thickness can be a few microns or less). Micro/nanostructuring of the semiconductor converter layer provides a way to overcome the high surface reflections (> 30% along the visible wavelength range) occurring for planar layers. Furthermore, the structuring is beneficial for improving the extraction of the emitted light. Other important advantages are the availability of appropriate materials (e.g., GaInP, AlGaInP, and InGaN) emitting in the visible spectral region and fabrication processes for structuring the material and integration. InGaN alloys are widely used in high efficiency blue LEDs but have limitations in terms of efficiency at other visible wavelengths^31^. However, different growth approaches are being investigated to improve the quality of InGaN alloys for the visible wavelengths^32–35^. On the other hand, using the maturity of GaN blue LEDs other colors can be provided by adding on color converting materials. These different approaches are mainly driven by the requirement of a common material platform for integration in advanced RGB LED technology. Currently, with III-nitride technology blue LEDs are well developed and green LEDs are continuously being improved in efficiencies^36,37^. Obtaining efficient red-light emission is one of the main bottlenecks in III-nitride based RGB LEDs^38,39^.

GaInP and AlGaInP alloys have been used in a wide range of optoelectronic applications involving efficient light emission or absorption in, e.g., solar cells^40^, window layers in solar cells^41^, LEDs^42^, and lasers^43^. Combining these materials with III-nitride LEDs could be of interest to cover the full visible range, for example by simply adding a red emitting (Al)GaInP layer on a blue or green LED and using a photoluminescence process to convert locally blue or green light to red light^28–30^. To reach the highest efficiency and compactness, the color converter must be of high optical quality and must efficiently absorb the incident light. For a given converter material, first reflection losses (typically > 30% for these materials) must be avoided to reach full absorption in the layer. Secondly, the converted light must be radiated efficiently into free space, i.e., avoiding light trapping/re-absorption in the converting layer. For example, this can be achieved by adding facets or introducing holes to reduce the effective index or to scatter light out of the emitting material^44–46^. Most often the excitation light is incident from a low-index medium, while light emission (from the converter) is from a high to low index medium. Besides this, the incident and emitted light wavelengths differ by as much as 100–200 nm. Thus, one can expect a compromise in the design of the converter layer to promote efficient absorption (by minimizing reflection losses) of the incident light and efficient out-coupling of light emitted (by photoluminescence) from the converter. Micro- and nanostructures have already been used to improve either light absorption^47^ or light extraction from semiconductor LEDs^48^, but less frequently optimizing both at the same time. Most of the studies concerning light extraction rely on experimental investigations, as rigorous simulation is time demanding and difficult to calibrate. On the other hand, optimization of absorption is relatively straightforward for simple geometric structures (e.g., periodic patterns), often performed using finite-difference time-domain (FDTD) simulations.

Bottom-up^49–51^ and top-down^52–57^ fabrication methods can be used to obtain (ordered) micro- and nanostructures in GaInP. In general, bottom-up approaches are based on growing the structures directly, for example by metal–organic vapor phase epitaxy (MOVPE) or solution synthesis. However, with these methods there are limitations in terms of the geometric shapes of the grown structures, lateral spacing, and material quality of ternary materials. On the other hand, top-down methods rely on pattern-transfer into the desired semiconductor layers using appropriate lithography and etching techniques, which are well developed for micro- and nanopatterning. In addition, for several III-V materials, including heterostructures, high quality epitaxial wafers are readily available, and the layer thicknesses and vertical sequence can be pre-designed. By appropriate methods, substrate-free micro- or nanostructures fabricated either by top-down or bottom-up approaches can be integrated in a transparent polymer or other type of films in order to obtain stand-alone films for specific applications such as color conversion and optical filters. Such a technology, as developed here, also enables to investigate the optical properties of the embedded nanostructures and their arrangements without substrate influences. Furthermore, such films with embedded structures can function as optical coatings for light-manipulation-function devices such as LEDs, solar cells, and photodetectors. For such coatings semiconductor nanowire (NW) or nanopillar (NP) arrays are excellent candidates since they can be fabricated by growth or by conventional pattern transfer methods. In the recent years, NWs/NPs have been extensively investigated for specific optical properties and device applications, such as broadband anti-reflection^47,58^, solar cells^59,60^, visible LEDs^33,34,48,61^, photodetectors^62^, and second-harmonic generation (SHG)^25,63^. Although NW/NP arrays can provide efficient in-coupling of light, efficient light absorption, and enhanced light extraction (compared to planar films) they are much less investigated as color converters.

**Figure 1:**
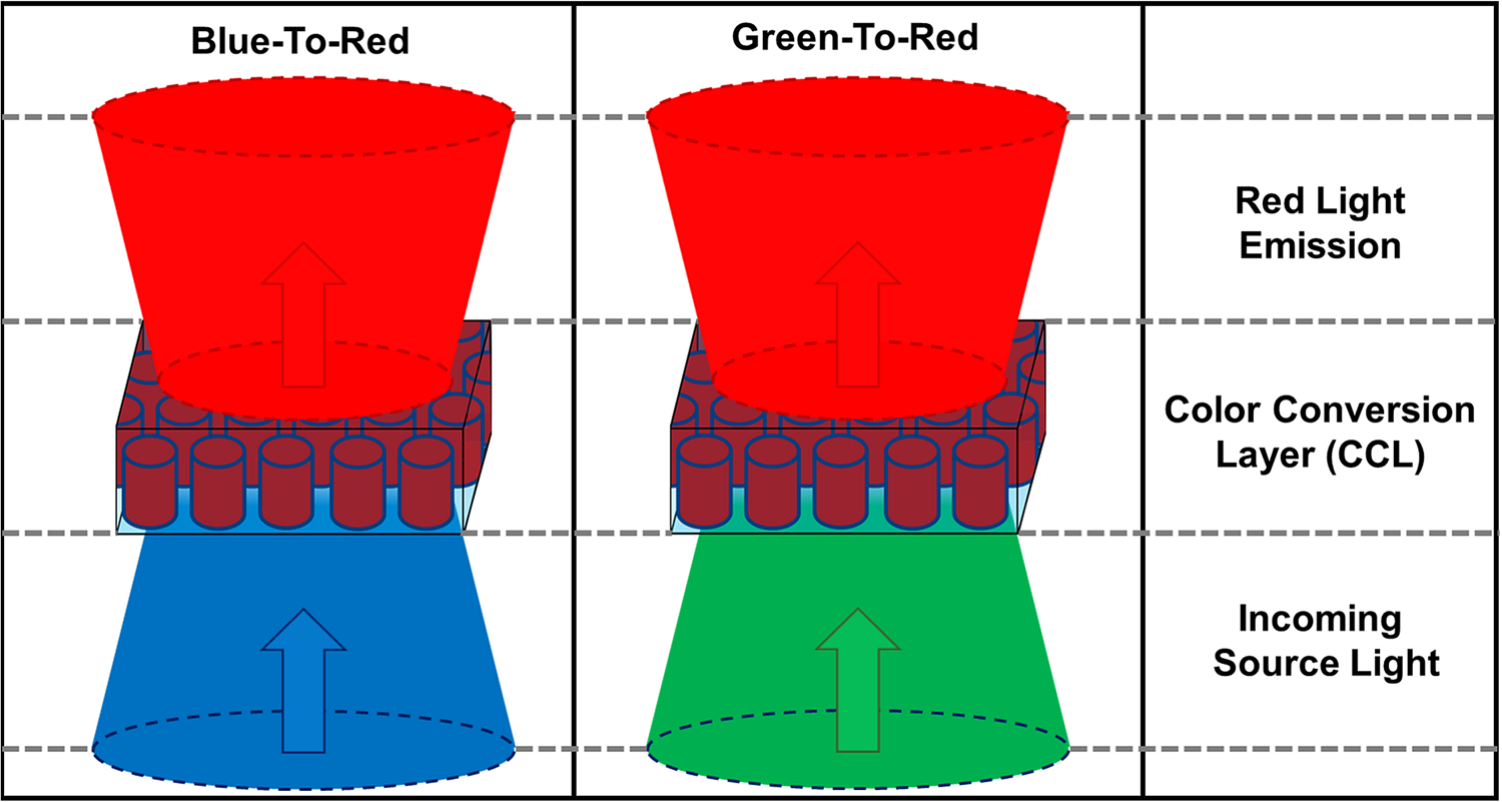
Schematics depicting the color conversion from blue-to-red and green-to-red based on (optimized) GaInP nanowire arrays embedded in a transparent film (color conversion layer (CCL)).

In this work, (tapered) Ga_0.51_In_0.49_P nanowire (NW) arrays are investigated for their color conversion properties for red light (~ 660 nm) emission. Tapered GaInP NW arrays were fabricated using colloidal lithography and dry etching. The specific composition of Ga_0.51_In_0.49_P has a direct bandgap, emits in the red, and is lattice matched to GaAs. The last aspect enables growth of desired stacks of GaInP/GaAs with less restrictions on layer thickness compared to strained layers, and fabrication of substrate-free GaInP structures by selective (sacrificial) etching of GaAs. The fabricated tapered GaInP NW arrays and (reference) micro-slabs are embedded in polydimethylsiloxane (PDMS) by removing them from the GaAs substrate and optically characterized by spectrophotometry and photoluminescence (PL). Such a configuration also neatly avoids any substrate influence (carrier or light leakage during optical studies). It is shown that (tapered) NW arrays improve the overall absorption compared to a simple slab, due to their anti-reflection properties. Furthermore, they show better light extraction by reducing optical trapping of the emitted light within the material. FDTD simulations are used to design the (tapered) GaInP NW arrays for minimum reflectance and maximum absorption of the source (blue and green) light. A top-down approach was used to fabricate tapered GaInP NW, micro-disk/square (MD/MS) arrays, where the (reference) MD/MSs mimic a GaInP ‘slab’. The fabricated GaInP structures are characterized for their reflectance, transmittance, and absorption using spectrophotometry, and by PL spectroscopy using UV (‘blue’; 405 nm) and green (532 nm) lasers as the excitation source. Schematic representations for the proposed color conversion layer for blue-to-red and green-to-red is shown in Fig. 1.

## Results and discussion

**Figure 2:**
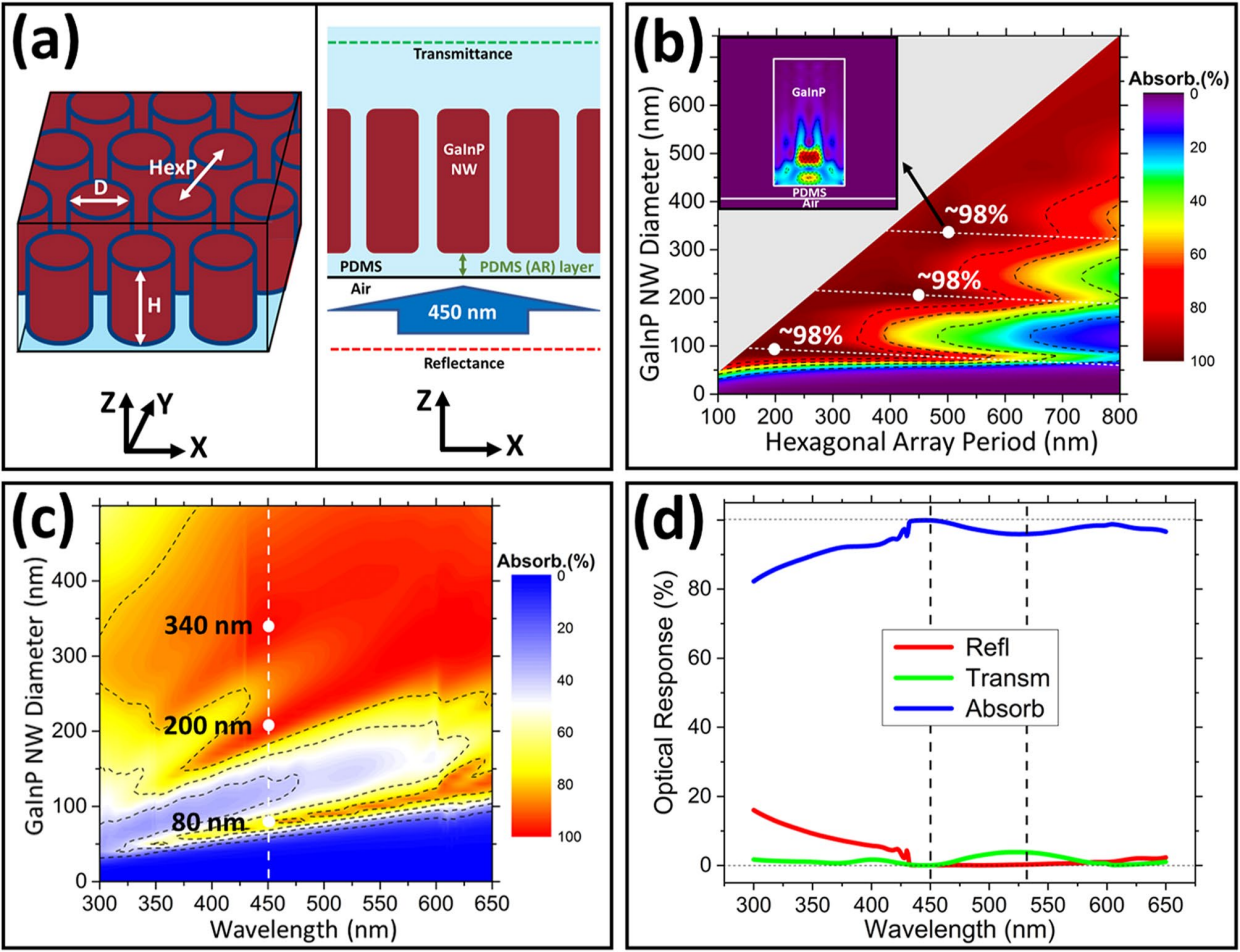
FDTD simulation results mapping the optimal geometrical parameters of the embedded GaInP NW arrays with a height (H) of 1 µm for 450 nm source light. (**a**) Schematics for the simulated embedded GaInP NW structures. (**b**) Contour plot showing the calculated absorbance (FoM) at 450 nm for the investigated diameters (Ds) and periods (HexPs). The white dots indicate the highest obtained FoMs. Inset: absorbed power distribution at 450 nm wavelength of incident light. (**c**) Contour plot for the FoM values for GaInP NW arrays with an H of 1 μm, HexP of 500 nm, and D swept between 0–500 nm. (**d**) The optical response (total reflectance, transmittance, and absorbance) for optimized GaInP NW arrays embedded in PDMS.

FDTD simulations, using a Lumerical tool, were utilized to design and investigate the optimal GaInP NW structures for maximum light absorption for above bandgap wavelengths in the range of 300–650 nm. Here, the focus is on absorption of 450 nm and 532 nm wavelength light, corresponding to blue and green LEDs. The absorption is considered as the figure of merit (FoM), where the highest value of the FoM shows the optimal parameters for the investigated geometries and spacing range. Figure 2a shows a schematic sketch of the simulated structures. The NW arrays have a height of 1 μm, and the hexagonal array period (HexP) and diameter (D) were varied between 100–800 and 0-HexP nm, respectively. A 100 nm thick PDMS film was used as an anti-reflection (AR) layer at the bottom of the embedded structures (see Fig. 2a). The height of 1 μm was chosen due to the penetration depth of blue (450 nm) and green (532 nm) light for the GaInP material used in this work, which are roughly ~ 600 and ~ 900 nm, respectively. The optical constants used for the GaInP material were taken from Schubert et al.^64^ For PDMS a refractive index of 1.4 is taken and the PDMS total slab thickness semi-infinite. For the wavelength range considered, spectrophotometry measurements show negligible absorption in the fabricated PDMS films (without embedded structures) and therefore taken as zero in the simulations. An E_x_-polarized plane wave source at normal incidence towards the air-PDMS interface is applied (see Fig. 2a) to mimic emission from a microcavity LED^65,66^. Influences due to polarization effects for these symmetric structures only arise for incidence angles > 10° and are therefore not relevant here. A reflectance monitor is used before the source and a transmittance monitor within the PDMS slab (see Fig. 2a). The transmittance monitor is placed in the PDMS to prevent possible Fabry-Pérot oscillations in the simulation when using a finite thickness of the PDMS slab and to limit the simulation time (for thick PDMS slabs). A reflectance loss of ~ 3% needs to be considered at the PDMS-air interface (above the embedded structures). Here we note that for a quantitative estimate of the reflectance loss, effects of light scattering by the NWs has to be evaluated. Figures 2b,c show the results for the FoM investigations for GaInP NW arrays embedded in PDMS; the incident light wavelength is 450 nm. Figure 2b shows a contour plot of the calculated FoM (‘Absorb.(%)’) as a function of the investigated HexPs and Ds for the NWs. FoM values > 98% are obtained for NW arrays at specific values of HexP and D: 200 and 90 nm, 450 and 210 nm, and 500 and 340 nm, respectively. The absorbed power distribution for one case is shown in the inset of Fig. 2b. Considering the nanopatterning method used in this work (colloidal lithography—CL), the most practical choice for the array period and NW diameter are 500 and 340 nm, respectively. Relatively large area (few mm^2^) close-packed monolayer coverage by spin coating can be obtained with 500 nm SiO_2_ colloidal particles and their diameter can be reduced by dry etching in a controlled manner^67^. Figure 2c shows the contour plot for the calculated spectrally dependent absorbance of the GaInP NW arrays as a function of the NW diameter (D); the NW height is 1 µm and the HexP is 500 nm. These results indicate three diameter values with the highest FoM values for incident light with a wavelength of 450 nm. The high absorbance for the diameters of 80, 200, and 340 nm are due to efficient coupling to HE_11_, HE_12_, and HE_13_ modes, respectively. These modes were identified using simulated field distribution plots, which are included in the Supplementary Information, Fig. S1.

Figure 2d shows the spectrally dependent optical response—total reflectance, transmittance, and absorbance—for an optimized GaInP NW array (HexP = 500 nm and D = 340 nm) embedded in PDMS. The optimized PDMS AR layer is 80 nm at the source side (incidence). This result clearly demonstrates close to 100% absorption at 450 nm can be obtained for the optimized GaInP NW array embedded in PDMS. Interestingly, the same structure also provides high absorbance (> 95%) in the green spectral region (around 532 nm). This can be improved to > 98% absorbance at 532 nm with an optimized value of 260 nm for the GaInP NW diameter. Simulation data regarding the FoM for GaInP NW array structures embedded in PDMS for other incident wavelengths relevant to this work (405 and 532 nm) are included in the Supplementary Information, Fig. S2. Due to the better in-coupling of light (nearly zero surface reflectance) at the specific wavelengths, the overall absorption is higher in the GaInP NW arrays embedded in PDMS compared to a planar (unstructured) slab with a surface PDMS AR layer (80 nm thick). At 450 nm, with an optimized 80 nm PDMS AR layer, surface reflectance of ~ 9% and ~ 0.05% are obtained for the GaInP slab and optimized NW array in PDMS, respectively. Additional advantages are that the NW array structures show a broader angular acceptance of the incident light and have a higher light extraction compared to an unstructured slab.

The calculated absorbance for the GaInP NW arrays in PDMS with tapered NW shapes similar to those fabricated in this work, indicates high absorbance > 96% can be obtained at 450 and 532 nm incident light wavelength. The average diameter (AvD) for the tapered NWs, defined as the arithmetic average of the top and bottom diameters, is in the range 230 to 290 nm. For these calculations, the PDMS AR layer was taken as 100 nm and is close to what is obtained in the fabricated samples. The simulated absorbance data for the tapered GaInP NW arrays in PDMS showing the influence of the NW shape are detailed in the Supplementary Information, Fig. S3. These results show that the overall absorbance is affected by the tapered shape. More importantly, for the average diameter range (230–300 nm) typically obtained in the fabricated tapered NWs the calculated absorbance (FoM) is comparable to the ideal case of cylindrical NW arrays (see Fig. 2). Below, we discuss the experimental results focusing on (tapered) GaInP NW fabrication and the optical characterization by spectrophotometry and PL.

**Figure 3:**
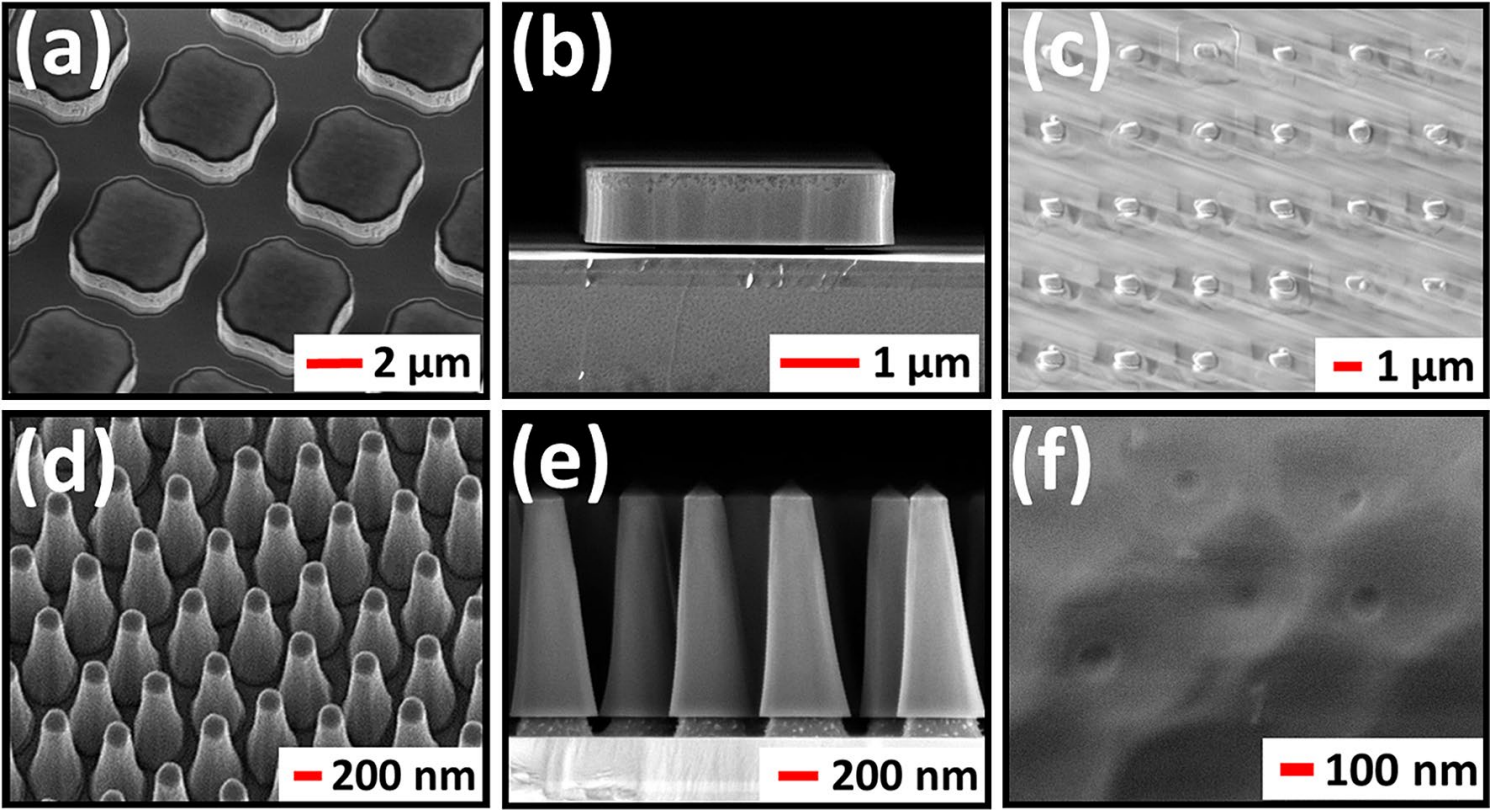
Scanning electron microscopy (SEM) images of the fabricated GaInP structures. (**a**–**c**): micro-square (MS) and (**d**–**f**) tapered nanowire (NW) arrays. Representative top view and cross-section (with partially etched GaAs sacrificial layer) images for the structures still on the substrate are included (**a**,**d**) and (**b**,**e**), respectively. Top view images for the respective structures embedded in PDMS (after peel-off) are shown in (**c**) and (**f**).

The GaInP micro- and nanostructures were fabricated from a 1 µm-thick nominally undoped Ga_0.51_In_0.49_P layer grown on (100) GaAs substrates, with an intermediate undoped GaAs buffer layer. Ga_0.51_In_0.49_P (further on referred as GaInP) is a direct bandgap material with band-edge PL in the red spectral region (~ 660 nm). A 1 μm-thick GaInP layer is sufficient for complete absorbance of both blue and green incident light. After pattern transfer the GaAs underneath the GaInP layer is sacrificially removed to generate substrate-free GaInP structures for embedding in PDMS. The first reason to remove the substrate is to favor carrier recombination in the GaInP, which has a higher bandgap than GaAs. The second reason is to be able to fabricate stand-alone color converting films from the substrate-free GaInP nano- and microstructures by embedding them in PDMS. Such films can also be handled easily for optical measurements. In Fig. 3 scanning electron microscopy (SEM) images are shown for the fabricated GaInP micro-square (MS) and tapered NW array structures. For the tapered NW arrays a combination of colloidal lithography (CL), based on the self-assembly of silicon dioxide (SiO_2_) colloidal spheres, and inductively coupled plasma reactive ion etching (ICP- RIE) was used. The tapered NW shape is most likely due to mask erosion during GaInP etching. For the microstructures, a combination of optical lithography and ICP-RIE was used. After dry etching an HF(5%) treatment is used to remove the SiO_2_ mask and subsequently a sacrificial wet etching process is used to partially remove the sacrificial GaAs layer (thickness of 100 nm) underneath the GaInP structures; thereby maintaining their original order. The structures were then embedded in a PDMS film and peeled-off from the substrate, thereby integrating the GaInP structures in a highly transparent film. Further details of the fabrication process are provided in the Methods section. Figures 3a–f show representative SEM images of the fabricated GaInP MS and tapered NW array structures still on the substrate and after embedding and peel-off in PDMS. Below the tapered GaInP NW arrays (see Fig. 3e) a partially etched GaAs layer (stem) is shown. This stem is further etched before embedding the GaInP structures in order to limit the size of the GaAs stem (diameter of ~ 50 to 100 nm). During the peel-off process of the PDMS from the original substrate, the embedded GaInP structures ‘break’ at the narrow GaAs stem. To ensure that only the GaInP structures are embedded in the PDMS any remnant GaAs at the bottom is removed by wet chemical etching (selective to GaAs). The MSs are ~ 3.5 µm in the lateral dimensions and are 1 μm in height; the square array period is 5 μm. The tapered NWs have a height of 1 μm, hexagonal array period of 500 nm, and the top and bottom diameters are ~ 150 and ~ 350 nm, respectively (AvD ≈ 250 nm). The size (length–width) of the MS structures was chosen to limit the GaAs sacrificial wet etch time and, additionally, for these structures a better control of the wet etch time was achieved in order to limit the GaAs stem below the GaInP structures before embedding. The gap between the MS structures was restricted due to the resolution (~ 1 μm) of the optical lithography tool. In addition to the tapered NW and MS arrays, 1 µm-thick GaInP micro-disks (MDs) were fabricated for PL measurements to mimic an unstructured slab. The MDs are ~ 20 μm in diameter, larger than the typical excitation spot size. Representative SEM images are shown in the Supplementary Information, Fig. S4.

**Figure 4:**
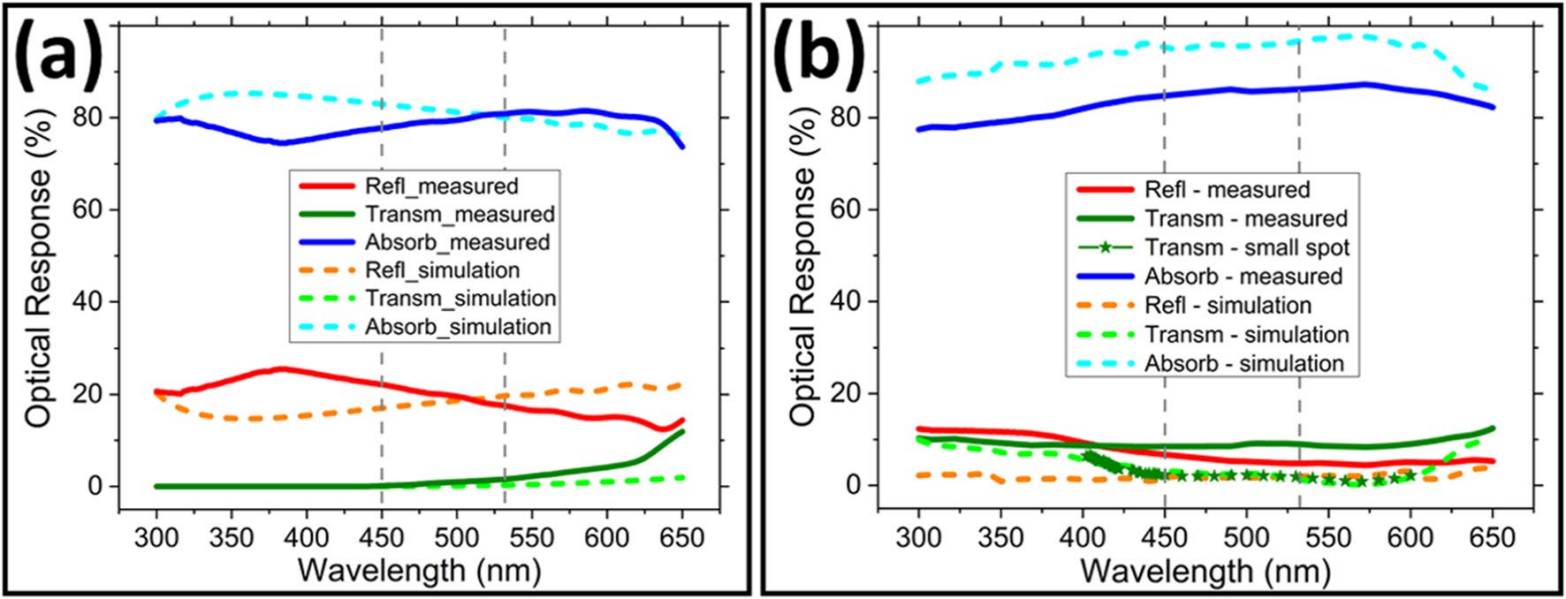
The optical properties (total reflectance, transmittance, and absorbance) for GaInP structures embedded in PDMS. (**a**) Simulated optical response for a 1 µm-thick semi-infinite thin film layer (slab). The ‘measured’ optical response for the micro-square (MS) arrays embedded in PDMS is included. This data is corrected for a fill factor of 100%. (**b**) Simulated and measured optical response for the tapered nanowire (NW) arrays embedded in PDMS. Measured transmittance using a small spot set-up is also included (dark green stars).

The reflectance (R) and transmittance (T) of the fabricated GaInP structures were measured by spectrophotometry in the wavelength range of 300–650 nm. The absorbance (A) was obtained as 100% − (R(%) + T(%)). The measured data together with the corresponding FDTD simulation data results are shown in Fig. 4. Figure 4a shows the simulated optical response for an infinite 1 µm-thick film (slab) embedded in PDMS. The simulation data shows that an absorbance of ~ 80% can be obtained for the embedded GaInP slab at source wavelengths of 450 and 532 nm; the absorbance is mainly limited by surface reflection which is ~ 20% at these wavelengths. The simulated result for the slab can be taken as a reference to compare the structured (MS or tapered NW array) converter layers. Figure 4a also shows the measured data for the MS array, corrected for the surface fill factor (FF) of the semiconductor (~ 49%). Although some light is transmitted through the gaps and the boundaries of the MSs also scatter light, approximating the MS array to a slab gives some qualitative information for comparison. As seen in Fig. 4a the measured and simulated data are within 10% in the full wavelength range. Figure 4b shows the measured and simulated optical response for the tapered GaInP NW (AvD ≈ 250 nm) arrays embedded in PDMS. The observed trends in the measured and simulated optical response are in very good qualitative agreement. The simulated data for the tapered NW arrays (modelled after the fabricated tapered NW shapes) shows very high absorbance, more than 95% at 450 and 532 nm source wavelengths. Correspondingly, the measured absorbance is ~ 85%. Since the tapered NW dimension and shape used in the simulations correspond to the fabricated ones, the lower measured absorbance is attributed to the non-homogeneous tapered NW (areal) coverage with open areas in the fabricated samples. This inhomogeneity is invariably observed in the CL procedure used. The largest area with monolayer hexagonally close-packed silica was ~ 2 mm^2^, while interrogation spot size in the spectrophotometer is about 2 to 4 mm^2^. Indeed, additional measurements using a small spot (~ 17 µm in diameter) transmittance measurement setup (green stars in Fig. 4b) confirmed the residual transmittance is only ~ 2 to 3% as predicted by the simulations for incident light with a wavelength of 450 and 532 nm, respectively. With improved CL procedure or by using nanoimprint lithography (NIL), uniform large area NW arrays can easily be fabricated.

Angular dependence of absorption in the NW arrays was investigated by FDTD simulations. The results show that the absorption properties of the NW arrays are better than that of a simple slab for all considered incidence angles (0–80°). Considering the NW array, we find high absorbance (> 95% for incidence angles up to ~ 50° from the surface normal) for both source wavelengths (450 and 532 nm). The results are shown in the Supplementary Information, Fig. S5. Analysis of the angular dependence for the emitted PL light by the NW array structures is complicated due to light scattering, light interference, waveguiding, and re-absorption effects and has not been considered in this work. A detailed investigation is necessary to address these issues.

**Figure 5:**
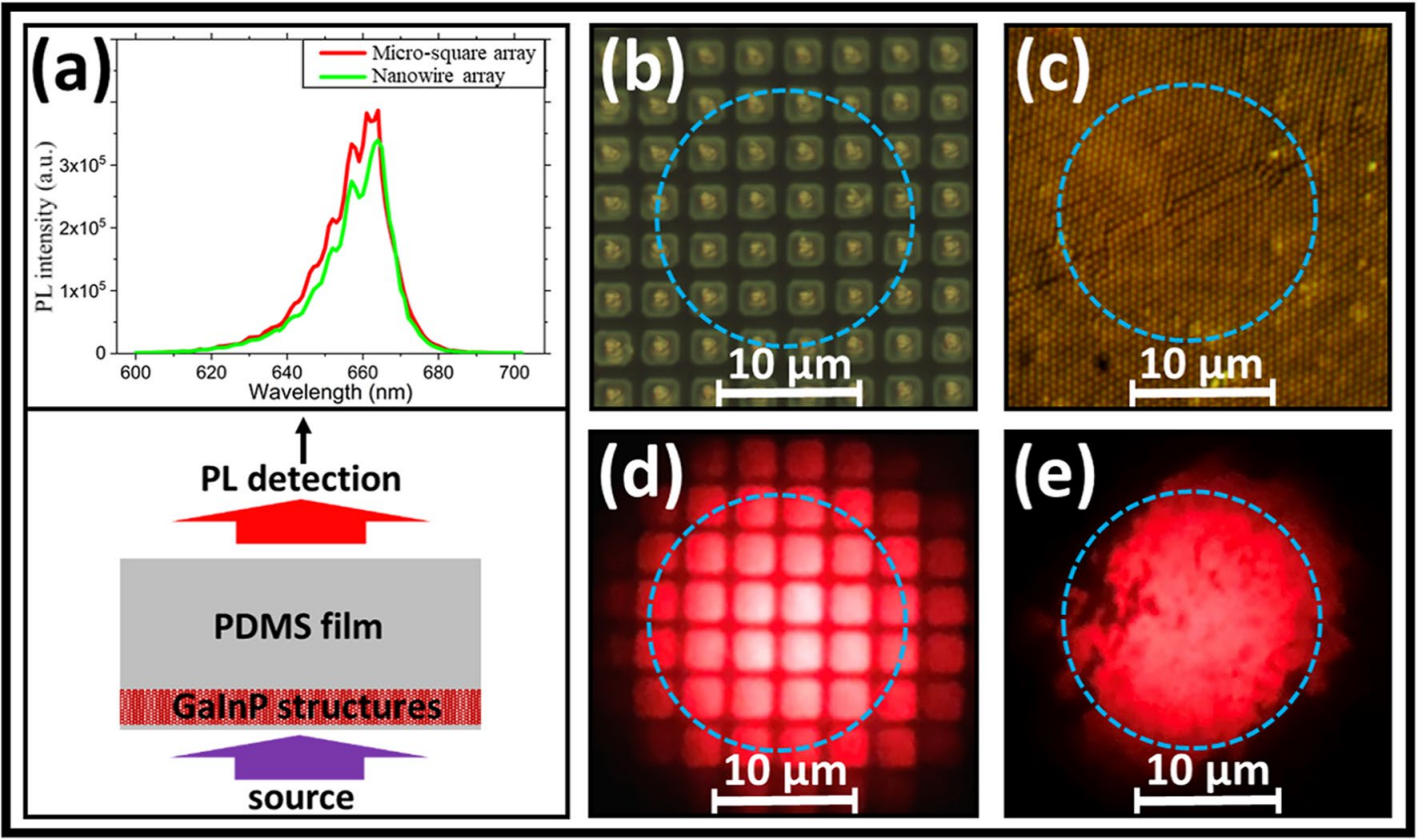
Color conversion results from 405 to ~ 660 nm for micro-square (MS) and tapered nanowire (NW) arrays embedded in PDMS with a spot size of ~ 17 μm and power density of ~ 2.65 μW/μm^2^. (**a**) Schematic indicating the sample orientation, direction for which the emitted PL light was measured, and the resulting PL spectra. The observed oscillations are due to interference effects due to the experimental configuration. Top view microscope images of the embedded MS and tapered NW array structures, respectively: before excitation (**b**,**c**) and under excitation (**d**,**e**), respectively. For the images in (**d**,**e**), a 405 nm notch filter was used before the camera objective.

Room-temperature PL experiments are performed for proof-of-principle demonstration of ‘color conversion’ using the embedded GaInP structures. Lasers with a source wavelength of 405 and 532 nm were used as the excitation sources. Although a 450 nm source would have been ideal, a 405 nm laser was used due to limitations in the available setup. The spectrophotometry results in Fig. 4b indicate a higher transmittance of the 405 nm source light (~ 9%) compared to the transmittance for a source light of 450 nm (~ 2%). However, a roughly similar portion (~ 1–2%) of reflected light is expected. The PL results for the MS and tapered NW arrays under 405 nm laser excitation are shown in Fig. 5. Corresponding PL results for a source wavelength of 532 nm is given in the Supplementary Information, Fig. S6. Figure 5a shows the schematic for the color conversion (PL) measurements and the measured PL spectra for the GaInP MS and tapered NW arrays embedded in PDMS films. The PL peak around 660 nm is observed for both the samples. Figures 5b,c show optical microscope images of the MS and tapered NW arrays embedded in PDMS, respectively. The 405 nm laser is focused by an objective (NA = 0.5) on the embedded GaInP structures, with a resulting spot size of ~ 17 µm; the power density was ~ 2.65 μW/μm^2^. After the sample, a notch filter is placed in order to cut-off any transmitted 405 nm light. However, representative camera images for both types of structures including both the source light (405 nm) and emitted PL light (~ 660 nm) are included in the Supplementary Information, Fig. S7; taken at a different spot than included in Fig. 5. The emitted light was collected by a microscope objective and subsequently imaged by a camera (Fig. 5d,e). Additionally, the PL spectrum was measured by a spectrophotometer at a similar spot for the embedded structures. The measured PL spectra show similar PL intensities (see Fig. 5a) for both types of structures. Although the tapered NW arrays have a much lower fill factor (FF≈24%), the PL intensity is comparable to the MS arrays (FF≈49%). The results for the color conversion from 532 to ~ 660 nm (Supplementary Information, Fig. S6), indicates a slightly higher PL intensity for the tapered NW arrays compared to the MS arrays for the same experimental conditions. These observations argue that tapered NW arrays perform better than MS arrays and slabs, with advantages of higher absorption of excitation light in the structure as well as enhanced light extraction. Additionally, PL measurements were performed for source wavelengths of 405 and 532 nm, respectively, for an isolated MD located on a quartz substrate; with the same excitation conditions as used for the MS and tapered NW array structures. The results for the slab-like MDs are included in the Supplementary Information, Fig. S8.

Substrate-free MDs were obtained by completely removing the sacrificial layer below the MDs by wet etching and retrieving the floating MDs from the solution directly on to a quartz substrate. The PL measurements were performed with the source light incident through the quartz substrate before illuminating the GaInP MD. The spot size was smaller than the MD diameter for all measurements. A light trapping effect within the MD layer can be inferred as shown by PL light guided within the layer and emitted out from the exposed sidewalls of the MD. These observations underline the limitations for light extraction for a GaInP slab/layer due to trapping influences.

Quantitative analysis of the color conversion efficiency of the GaInP based color converter films requires extensive measurements as well as detailed investigations of surface passivation of the etched tapered GaInP NW surfaces^68–70^, which are beyond the scope of the present work. Other lithography methods such as nanoimprint lithography (NIL) may be more suitable to obtain scalable and uniform coverage of embedded (tapered) NW arrays. Both fabrication and material improvements are necessary for integration of the GaInP NW arrays as color converters in blue and green LED technology.

Quantitative determination of the internal quantum efficiency (IQE) requires extensive investigations. Approaches often used include “estimation” of the radiative lifetimes by cooling the sample to very low temperatures (~ 10 K or lower) or by using the low temperature PL yield as a reference (assuming at low temperatures the IQE is 100%). Both these experiments were difficult with the present samples since they were embedded in PDMS and the in-homogenous spatial coverage of the tapered NW arrays. To address this issue, we make use of the PL decay times determined at room temperature and compare (as a ratio) the IQE of the different structures. Although, this approach is not rigorous it can be used for qualitative comparison of the material quality. We use the substrate-free slabs (micro-disk – MD) as the reference, representing an unstructured GaInP layer. These are sufficiently thick to absorb blue and green light. Room-temperature time-resolved photoluminescence (TRPL) measurements of the fabricated GaInP structures (as-processed) showed best lifetimes for the GaInP MD and lowest for the tapered NW array^70^. Representative TRPL data for the relevant structures are included in the Supplementary Information, Fig. S9. The PL decay times can be determined by fitting the TRPL data with a double exponential decay. The dominant PL decay times are ~ 3 ns, ~ 2.5 ns, and ~ 0.5 ns for the MDs, MS arrays, and NW arrays, respectively. For the NW arrays surface recombination dominates, as expected from their geometric dimensions. Reported values for the radiative lifetimes in GaInP with similar composition are in the range ~ 10 to 40 ns^71,72^. Following Schubert^30^, the IQE is given by the ratio of probabilities of radiative (1/τ_R_) and total recombination (1/τ = 1/(1/τ_R_ + 1/τ_NR_)), where τ_R_ and τ_NR_ are the radiative and non-radiative recombination lifetimes. We make the assumption that for all the processed structures τ_R_ >  > τ_NR_^71,72^. Consequently, the IQE for the processed structures can be obtained as the ratio of the non-radiative and radiative lifetimes (τ_NR_/τ_R_). Using this we can compare the IQEs of the different fabricated structures by determining the ratio of the IQEs related to the MD structure. Using the determined decay times, the IQEs of the MS array and the NW array are estimated to be ~ 80% and ~ 20% of that of the MD. We believe, detailed investigations on surface passivation methods are required to improve these values. Even though the tapered NW arrays show a lower IQE compared to MDs and MS arrays, they have the highest average PL intensity for the same measurement conditions. The higher PL intensity is attributed to a better in-coupling of the source light and better out-coupling of the PL light.

Besides improvements on passivation and coverage, further improvements can be obtained by utilizing GaInP/AlGaInP quantum well (QW) structuring. These QWs can be used to tune the emitted color to a more desired emitting wavelength of red light (e.g., wavelengths between ~ 620 and 640 nm). These QWs additionally can improve the IQE for color conversion, color rendering for RGB-displays, as well as the light extraction efficiency (since the QW emission is not re-absorbed by the barrier and emitting dipoles are mainly in-plane dipoles for GaInP/AlGaInP QW structures). The optimal location of the QW layers in the NW array structures can be related to the absorbed power distribution in the NW array structures, in order to maximize the carrier collection in the QW region. Simulations of the absorbed power (see for example the inset of Fig. 2b) indicate that most of the light is absorbed at a penetration depth of ~ 200 nm within the NW (most likely due to a multimode imaging effect^73^). Thus, placing QWs close to this position could help improving the IQE. Initial simulations (Supplementary Information, Fig. S10) done for light extraction from GaInP/AlGaInP QW-based NW array structures confirmed that high light extraction efficiencies can be achieved.

Color conversion films consisting of embedded GaInP NW array structures have advantages over the currently used color conversion films. For example, (a) they provide both high absorption of the source light (blue and green) as well as high light extraction of the emitted PL (red) light, (b) the structures can be integrated within the current RGB technology, and (c) directional emission of the converted light, *in principle*, can be obtained by tuning the geometry and spacing of the structures^74,75^. On the other hand, with surface structuring process-induced damage and surface states degrade the material quality, which is a disadvantage. Fabrication processes have to be optimized for minimizing damage and additional surface passivation steps may be required. While quantum dots (Q-dots) are successfully used for color conversion in conventional RGB technology, currently used color conversion films based on Q-dots require relatively thick layers (> 5 μm) to completely remove the source light (e.g., blue light)^19^. Thus, for µ-LED applications technological improvements to make the Q-dot layers thinner (~ 1 µm) to completely absorb the source light or to add separate filters to block the source light are seen as necessary^19^.

## Conclusion

This work shows that GaInP nanowire array structures embedded in a highly transparent film are interesting for color conversion applications. Absorption of blue and green incident light close to ~ 100% is possible for optimized cylindrical nanowire array structures. Nanowire array structures are able to show better color conversion properties due to a better in-coupling of the incident source light and a better out-coupling of the emitted light compared to a simple GaInP slab or film. Tapered GaInP nanowire arrays and (reference) microstructures embedded in PDMS were fabricated by a top-down method from high-quality epitaxially grown GaInP/GaAs stack layers on a GaAs substrate. The GaInP microstructures were used to mimic a GaInP slab. A combination of colloidal lithography (tapered nanowire arrays) or optical lithography (microstructures) with ICP-RIE, sacrificial GaAs layer wet etching, and PDMS embedding were used to obtain substrate-free GaInP structures embedded in a transparent film (PDMS). The absorption properties of the GaInP structures were characterized by spectrophotometry. Room-temperature photoluminescence was used as a proof-of-principle in order to demonstrate their color conversion to red light emission. This was demonstrated from UV (’blue’; 405 nm) and green (532 nm) to red (~ 660 nm) light emission by a photoluminescence process, thereby confirming the light conversion process for red light emission.

## Methods

### GaInP/GaAs stack layers

The epitaxially grown GaInP/GaAs stack layers were obtained from ENT S. A., Poland. The stack layer consists of the following top layer sequence: 1 μm GaInP/100 nm GaAs/250 nm GaInP/GaAs substrate. The top 1 μm GaInP layer was utilized to obtain the GaInP structures; therefore, resulting in structure heights of 1 μm. The 100 nm GaAs below this layer was used as a sacrificial layer in order to obtain substrate-free structures. The photoluminescence (PL) emission wavelength along the original wafer varies between ~ 657 and ~662 nm due to non-uniformity in the wafer. Wafer pieces of ~ 2 × 2 cm were used for the processing steps, resulting in slightly different PL properties from piece to piece.

### Colloidal lithography

For the colloidal lithography (CL), a SiO_2_ colloid solution was used obtained from Sigma Aldrich with average sphere sizes of ~ 500 nm. On top of the 1 μm GaInP layer a thin layer (~ 55 nm) of SiO_2_ was deposited by plasma-enhanced chemical vapor deposition (PECVD) with the deposition parameters: 2%SiH_4_/N_2_ at 710 sccm, N_2_O at 425 sccm, temperature of 300 °C, RF power of 20 W, pressure of 800 mTorr, and planar (calibrated) deposition rate of ~ 1.1 nm/s. This SiO_2_ layer increases the wettability of the surface for the CL and prevents undesired etching of the top layer below the SiO_2_ colloids^67^. The colloidal solution was then spin coated using a three-step process with spin-off speeds of 200, 1000, and 2000 rpm for 30 s, 2 min, and 10 s, respectively, with a spin-on speed of 100 rpm/s. This resulted in close-packed hexagonal array patches of SiO_2_ colloids with a homogeneous coverage of several mm^2^ and where the period is determined by the initial colloid size. Reactive ion etching (RIE; CHF_3_ flow of 25 sccm, RF power of 100 W, pressure of 50 mTorr, and an average diameter size reduction rate of ~ 25 nm/min) was used to size reduce the SiO_2_ colloids to the desired diameter size, where the size-reduced SiO_2_ colloids function as an etch mask for GaInP. Simultaneously the thin PECVD layer is etched when exposed, resulting in a slightly larger diameter disk shape below the SiO_2_ colloids.

### Optical lithography

Optical lithography was used to fabricate the micro-disk (MD) and micro-square (MS) arrays. For the MD arrays a (chrome) mask was used with MD patterns with a diameter of 20 μm and square array period of 30 μm. For the MS arrays, a (chrome) mask was used with micro-square patterns with a width/length of 3.5 μm and square period of 5 μm. A 300 nm SiO_2_ PECVD layer was first deposited on top of the 1 μm GaInP layer, then treated with hexamethyldisilazane (HMDS), and finally a ~ 1.8 μm thick photoresist layer of the positive photoresist MEGAPOSIT SPR700-1.8 (Rohm and Haas Electronic Materials) was deposited by spin coating with a spin-off speed of 5000 rpm for 1 min. A Mask Aligner MA6/BA6 Karl Suss optical lithography tool was used in vacuum contact mode, with an UV light (405 nm) exposure time of 7 s. After exposure, the sample was developed using MICROPOSIT MF CD-26 DEVELOPER for ~ 50 s. This treatment removes the exposed areas, resulting in the MD and MS array photoresist structures on top of the PECVD SiO_2_ layer. The exposed SiO_2_ layer was then removed by RIE, transferring the MD and MS array structures into the SiO_2_. The obtained SiO_2_ microstructures were used as an etch mask for GaInP, for which the photoresist is removed before ICP-RIE.

### ICP-RIE

An inductively coupled plasma reactive ion etching (ICP-RIE) process is used for the top-down fabrication of the GaInP structures by using a Cl_2_:H_2_:CH_4_-based chemistry (Cl_2_ flow of 9 sccm, H_2_ flow of 5.5 sccm, CH_4_ flow of 7.5 sccm, platen temperature of 60 °C, set pressure of 4 mTorr, ICP power of 1 kW, RF power of 100 W, and (calibrated) etch rate of ~ 170 nm/min). A selectivity between the GaInP:SiO_2_ mask in the order of ~ 4–5 was observed, resulting in tapered side-walls.

### Wet etch

After the ICP-RIE the SiO_2_ etch mask is removed by an HF(5%) treatment for 5 min. A wet etch process based on H_3_PO_4_:H_2_O_2_:H_2_O (3:1:25; etch rate of ~ 300 nm/min) is used to partially etch the GaAs sacrificial layer underneath the ICP-RIE structures. A small GaAs sacrificial layer contact point (stem) is kept below the structures in order to maintain the (array) order of the structures. The wet etch time has been chosen accordingly to this.

### PDMS embedding

After the partial wet etch of the GaAs layer underneath the GaInP structures, polydimethylsiloxane (PDMS; Dow Corning, Sylgard 184 Silicone Elastomer) embedding and lift-off is used to transfer the GaInP structures into a transparent film. This results in a 100 nm PDMS layer below the GaInP structures still on the substrate. However, it should be noted that this PDMS layer is absent at the locations where the GaAs stem was left after the partial GaAs wet etching step. An additional GaAs wet etch step using the H_3_PO_4_:H_2_O_2_:H_2_O chemistry was applied to remove any residual GaAs after the PDMS peel-off. The total PDMS thickness of the fabricated PDMS slab is ~ 1.5–2 mm.

### Simulations

Finite-difference time-domain (FDTD) simulations (Lumerical tool) were used for the modeling and optical simulations. The optical constants for GaInP were taken from Schubert et al.^64^. For PDMS a constant refractive index of 1.4 was considered and non-absorbing within the investigated wavelength spectrum (300–650 nm).

### Spectrophotometry

The measured total reflectance (R) and transmittance (T) spectra were obtained using a Lambda 950 UV/Vis/NIR spectrophotometer equipped with an integrated sphere and a spot size in the order of ~ 2 to 4 mm^2^. The absorbance (A) spectra were obtained by using A = 100% − R(%) − T(%).

### Scanning electron microscopy

A Zeiss Ultra55 scanning electron microscopy (SEM) tool was used to image the topography (top view & cross-section images) of the fabricated structures.

### PL measurements

A continuous wave (CW) laser was illuminated on the sample with two different excitation wavelengths: 405 and 532 nm. The original power was 0.6 and 1 mW for the wavelengths of 405 and 532 nm, respectively. The laser first passes a cold mirror (acting as a dichroic mirror) and is focused by an objective (50x) on the embedded GaInP structures. This results in a spot size of ~ 17 and ~ 11 μm for the 405 and 532 nm source light, respectively. After the sample, a notch filter is used to cut-off any transmitted source light. The emitted light from the GaInP structures is then collected by a microscope objective (NA = 0.5). The emitted red light was finally imaged (microscope objective 5x) using a camera or launched into a multimode fiber (microscope objective 50x; detection spot size of ~ 14 μm) in order to measure its spectrum with a spectrophotometer, having a minimum detection wavelength of 550 nm.

## Supplementary Information

Supplementary information.

## Abbreviations

A: Absorbance
AlGaInP: Aluminum gallium indium phosphide
CCL: Color conversion layer
CL: Colloidal lithography
CW: Continuous wave
D: Diameter
E-field: Electric field
FDTD: Finite-difference time-domain
FF: Fill factor
FoM: Figure of merit
GaAs: Gallium arsenide
GaInP: Gallium indium phosphide
H: Height
HexP: Hexagonal array period
H-field: Magnetic field
HF: Hydrofluoric acid
ICP-RIE: Inductively coupled plasma reactive ion etching
InGaN: Indium gallium nitride
IQE: Internal quantum efficiency
LED: Light emitting diode
MD: Micro-disk
MS: Micro-square
NA: Numerical aperture
NIL: Nanoimprint lithography
NIR: Near-infrared
NP: Nanopillar
NW: Nanowire
PL: Photoluminescence
PDMS: Polydimethylsiloxane
Q-dot: Quantum dot
QW: Quantum well
R: Reflectance
RGB: Red–green–blue
RIE: Reactive ion etching
SEM: Scanning electron microscopy
SiO_2_: Silicon dioxide
T: Transmittance
UV: Ultraviolet
vis: Visible
WCP: Wavelength conversion particles/polymers
