# Supplementary material for: GaInP nanowire arrays for color conversion applications
Source: Sci Rep. 2020 Dec 22;10:22368. doi: 10.1038/s41598-020-79498-2 (PMC7755895; doi:10.1038/s41598-020-79498-2)
Supplement: Supplementary file 1 — Supplementary information. [file 41598_2020_79498_MOESM1_ESM.pdf]

Supplementary Information

for

GaInP Nanowire Arrays for Color Conversion Applications

Dennis Visser[1,], Yohan Désières[1,2], Marcin Swillo[3], Eleonora De Luca[3], and Srinivasan Anand[1]*

[1]Department of Applied Physics, KTH Royal Institute of Technology, Electrum 229, SE-164 40 Kista, Sweden
[2]University Grenoble Alpes, CEA, LETI, MINATEC Campus, F-38054 Grenoble, France
[3]Department of Applied Physics, KTH Royal Institute of Technology, Roslagstullsbacken 21, SE-106 91 Stockholm, Sweden
*dvisser@kth.se

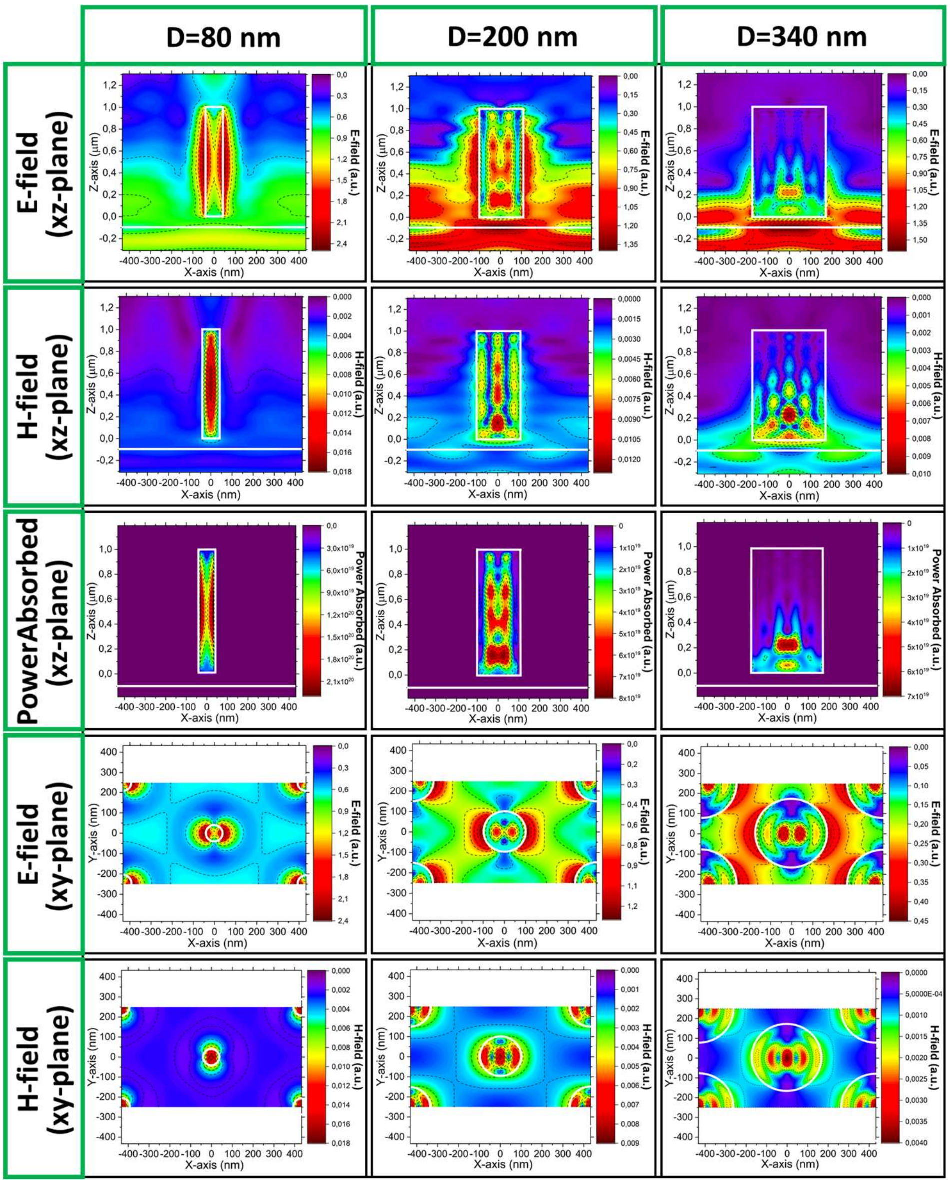

Figure S1. FDTD simulation data showing the cross-sectional E- and H-field distribution profiles and the absorbed power in cylindrical GaInP nanowires (NWs) embedded in PDMS. The vertical (xz) and horizontal (xy) cross-sectional profiles are from the respective central planes of the NW. The data is shown for three different NW diameters: 80, 200, and 340 nm; the array period and pillar height are 500 nm and 1 μm, respectively. The field profiles in the NWs indicate HE_{11}, HE_{12}, and HE_{13} modes for the NWs with a diameter of 80, 200, and 340 nm, respectively. An E_x-polarized plane wave source (light wavelength of 450 nm) incident along the z direction is used. The plots show the sum of the incident and scattered field components.

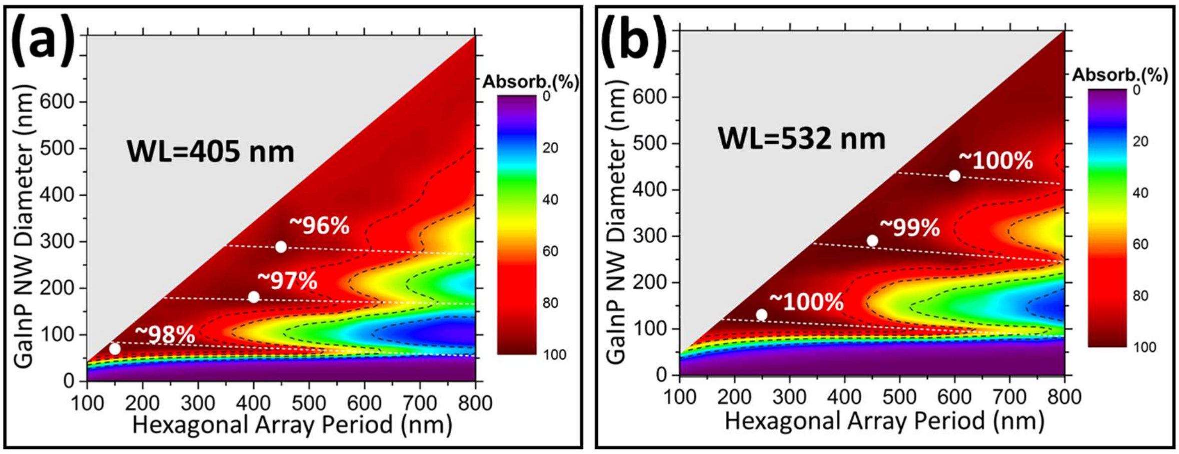

Figure S2. Contour plots showing the calculated absorbance (used as a figure of merit - FoM) for the GaInP NW hexagonal arrays embedded in PDMS as a function of the NW diameter (D) and period (HexP) for source wavelengths of (a) 405 and (b) 532 nm, respectively. The NW height is 1 µm. At both wavelengths, 405 and 532 nm, near complete absorption in the GaInP NWs is possible by appropriate choice of NW diameter and period.

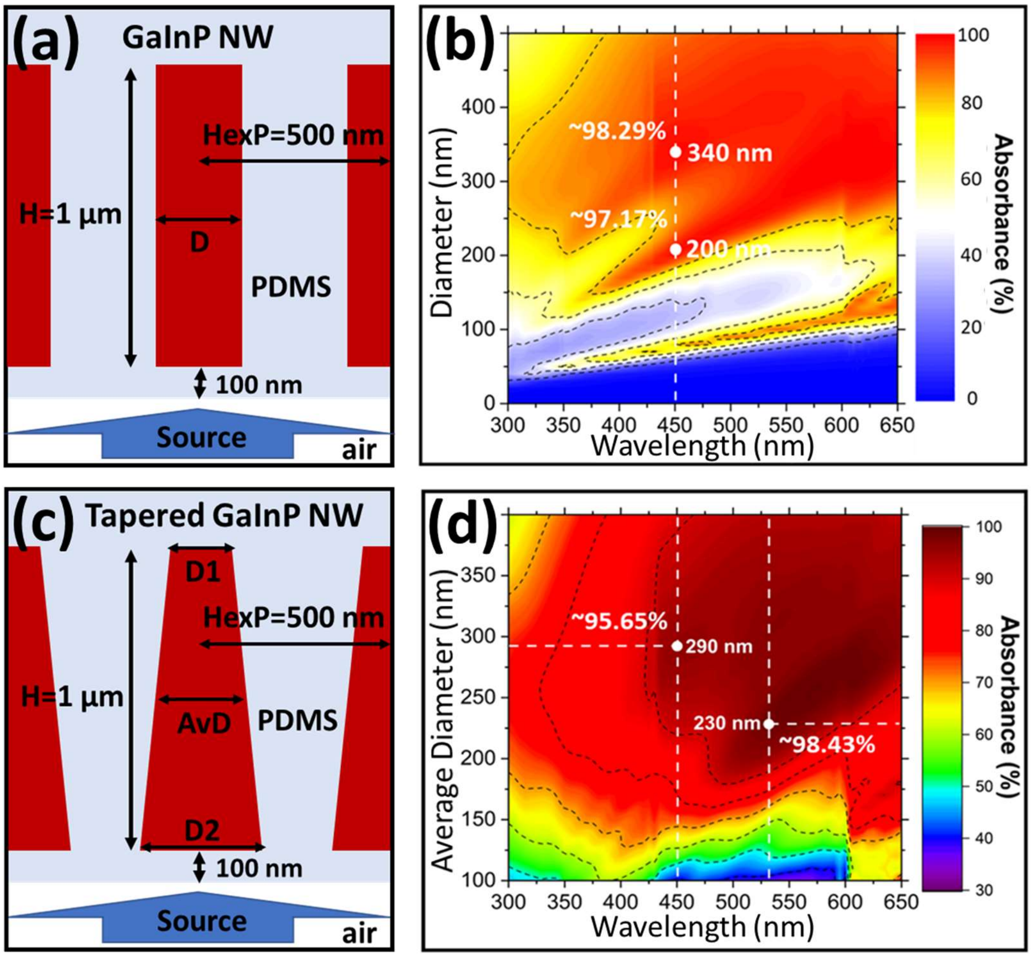

Figure S3. FDTD simulations showing the effect of the (tapered) nanowire (NW) geometry (shape and lateral dimensions) on the wavelength dependent absorbance of the tapered GaInP NW arrays embedded in PDMS. The hexagonal array period is 500 nm and the (tapered) NW height is 1 µm. (a) and (b) - schematic sketch and the calculated absorbance as a function of the diameter for NW arrays, respectively. (c) and (d) – schematic sketch and calculated absorbance for tapered NWs as a function of the average diameter. For the cylindrical NWs (b) the NW diameter (D) is swept from 0-500 nm with 10 nm steps. For the tapered NWs (d), the top diameter (D1) is swept from 0-300 nm with 10 nm steps. Simultaneously, the bottom (base) diameter D2 is varied such that

'D2=200+D1 nm', which maintains the same taper angle (like the fabricated structures). When D1=300 nm, D2 is equal to the array period (500 nm). Accordingly, the average diameter (AvD) is equal to AvD=(D1+D2)/2=(100+D1) nm. In (b) and (d) the (tapered) NW diameter parameter values for the highest absorbance are indicated for the corresponding source wavelengths. The absorbance (A) has been calculated from the simulated total reflectance (R) and transmittance (T) of the GaInP NW array embedded in PDMS, using the relation 'A(%)=R(%)+T(%)'. A plane wave source (E_x) at normal incidence from air to PDMS (with the embedded structures) is used.

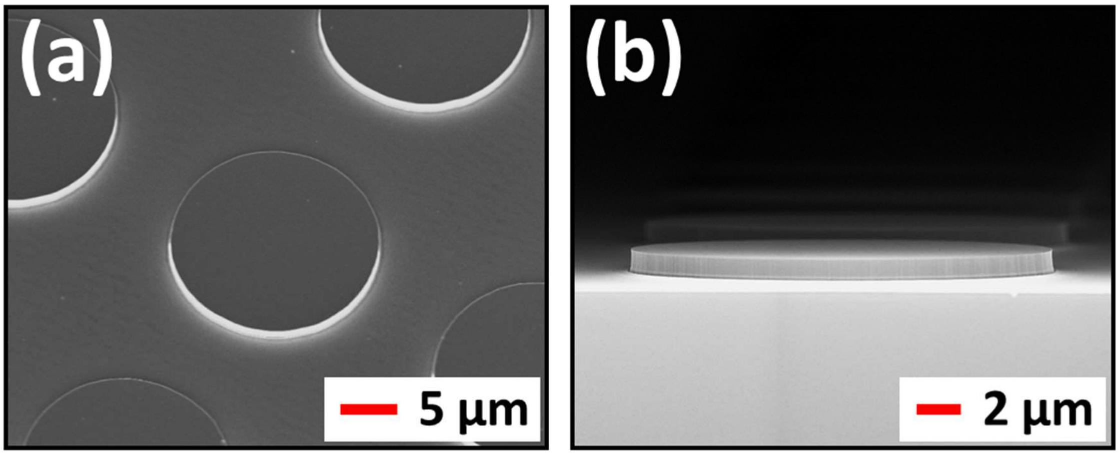

Figure S4. Scanning electron microscopy (SEM) images for the fabricated micro-disk (MD) arrays still on the original substrate (after ICP-RIE): (a) tilted top view and (b) cross-sectional view.

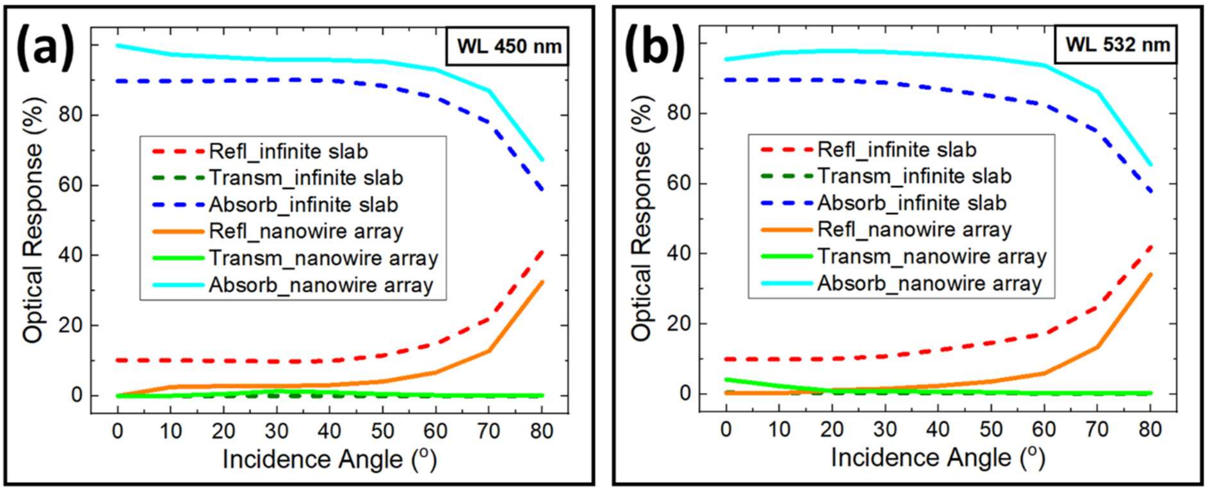

Figure S5. FDTD simulations regarding the optical response (total reflectance (Refl), transmittance (Transm), and absorbance (Absorb)) for an embedded infinite GaInP slab and GaInP nanowire arrays for a source wavelength of (a) 450 nm and (b) 532 nm, and for incidence angles varying between 0-80°. The infinite slab has a height of 1 μm and a PDMS AR layer of 80 nm. The GaInP nanowires have a height of 1 μm, diameter of 340 nm, hexagonal array period of 500 nm, and PDMS AR layer of 80 nm. A plane wave (BFAST) source has been used where non-polarized light has been considered by averaging the s- and p-polarized light. The reflectance and transmittance monitors were placed in the same way as depicted in Fig. 2a of the manuscript.

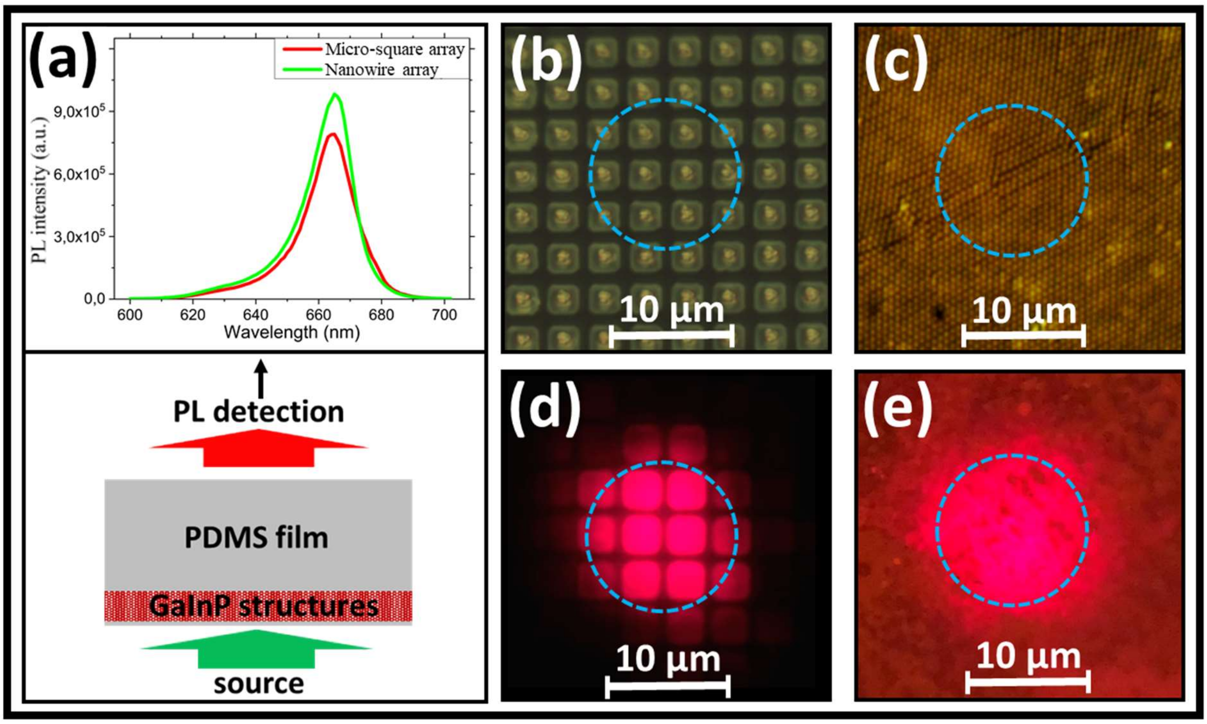

Figure S6. Color conversion by GaInP structures embedded in PDMS films via absorption of 532 nm (green light) excitation source and band-edge red light emission (photoluminescence) at ~660 nm. (a) Measured PL spectra from the GaInP micro-square (MS) and tapered nanowire (NW) arrays embedded in PDMS films; a schematic sketch of the measurement configuration is also shown. Top view microscope images of the MS and tapered NW arrays in PDMS, respectively: (b) and (c) are taken before and (d) and (e) under illumination with 532 nm laser light. The excited region (~11 µm) is indicated by the dashed circle. The images in (d) and (e) show only the converted light; the laser light is filtered out using a notch filter.

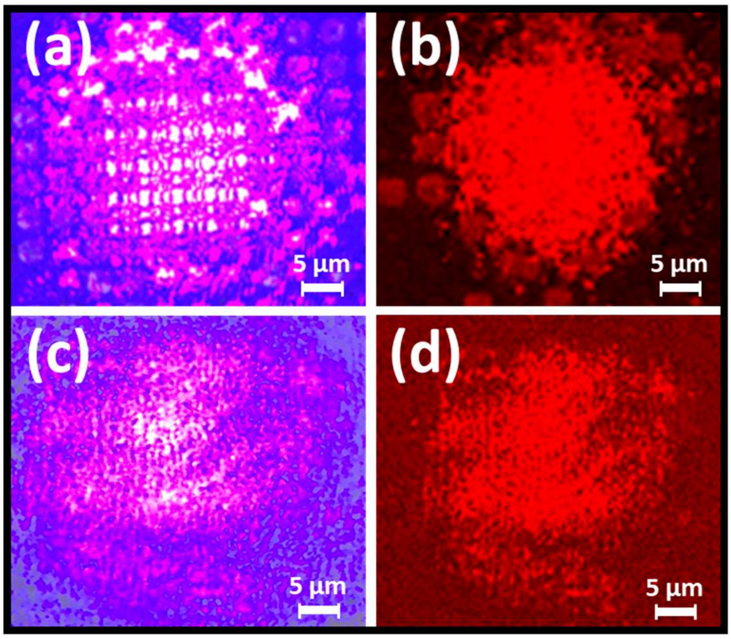

Figure S7. Color microscope images of the GaInP MS ((a) and (b)) and tapered NW ((c) and (d)) arrays in PDMS under 405 nm laser excitation. The spot size was ~30 µm. The images in (a) & (c) include the source light together with the emitted PL light (~660 nm). (b) & (d) show the respective images after applying a red filter (by an image analysis software; Picture & AVI converter v3.6). The observed spatial variations in the images are primarily due to inhomogeneities in spatial distribution of the GaInP structures in the imaged regions and light scattering effects.

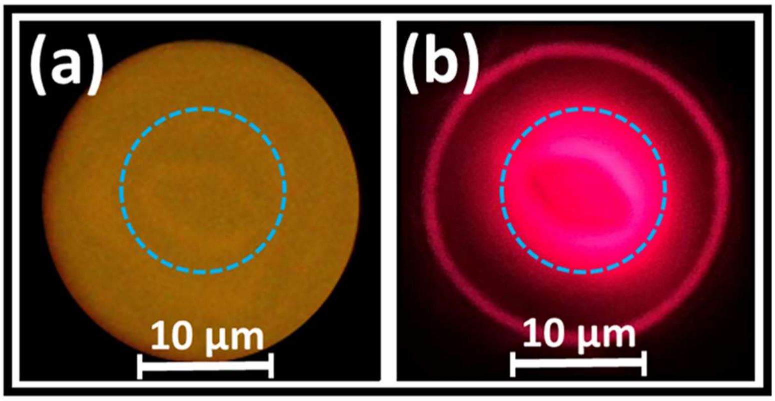

Figure S8. Color conversion from a GaInP slab (micro-disk (MD)) via absorption of above bandgap light and band-edge light emission. The GaInP MD is released from the GaAs substrate by selective etching and deposited onto a quartz carrier. The GaInP MD (1 µm thick) is sufficiently large in diameter (20 µm) compared to the typical excitation spot sizes (~11 µm). (a) Top view microscopy image (reflection mode) of the GaInP MD, taken with a white light source. (b) Representative top view microscopy images showing the red light emitted by the GaInP MD excited by 532 nm excitation. A similar image was obtained for the 405 nm source light. The observed ring of red light in (b) is due to the trapped PL light in the layer scattered from the edges.

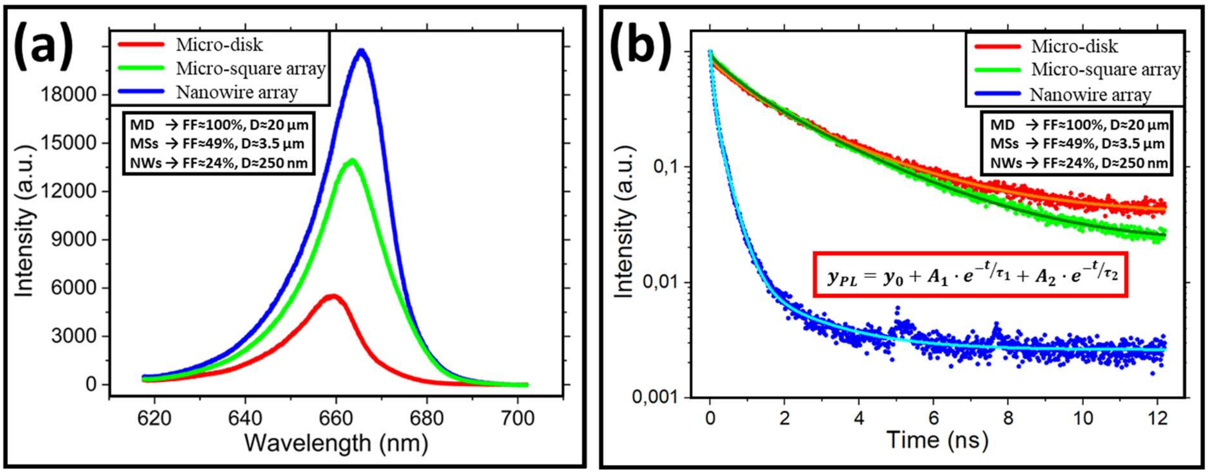

Figure S9. (a) and (b) The average photoluminescence (PL) and normalized time-resolved PL (TRPL) results, respectively, for the embedded micro-disk (MD), micro-square (MS) arrays, and tapered nanowire (NW) arrays in PDMS for a spot size of ~20 µm, average power density of ~570 µW/µm^2, and light source wavelength of 532 nm. The variations in the peak positions in (a), are due to non-uniformity in the original GaInP-GaAs wafer. The insets indicate the relative fill factors (FF) and sizes (D) of the embedded structures. The measured TRPL data was fitted with a two exponential decay function (see inset (b)). For the MD PL decay times were obtained in the order $\tau_1 \approx 3$ ns and $\tau_2 \approx 0.6$ ns, and for the MS arrays in the order of $\tau_1 \approx 2.5$ ns and $\tau_2 \approx 0.6$ ns. The shorter decay time is due to surface recombination and the longer decay time due to bulk recombination. For the tapered NW arrays a PL decay time of $\tau_1 \approx 0.5$ ns was determined when fitting with a single exponent ($A_2=0$). [1]

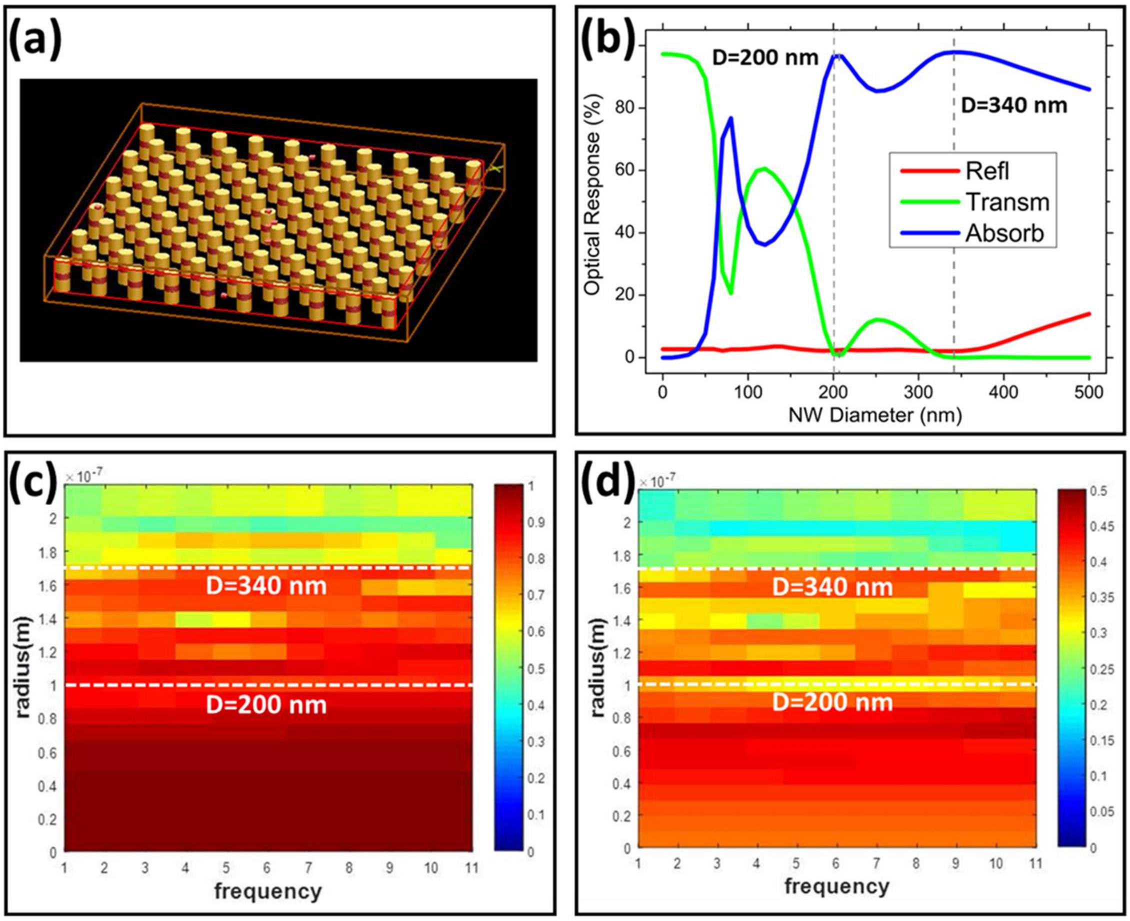

Figure S10. FDTD simulations for the light extraction efficiency (LEE) for source wavelengths varying between 620-640 nm, emitted from a GaInP/AlGaInP QW-based nanowire (NW) array structure embedded in PDMS. The structures have a height of 1 µm, hexagonal array period of 500 nm, and diameters varying between 0-500 nm. In (a) the schematic/model is shown for the simulated NW array structures. Many dipoles with different orientation were placed in the middle of one NW to mimic the quantum well(s) (QW(s)). In the QW region absorption (k) is taken as 0.02 to consider reabsorption of the QW(s). The NW portion outside the QW region is taken as non-absorbing and with a RI of 3.6. In (b) the optical response is shown for the total reflectance, transmittance, and absorbance for GaInP NWs with diameters varying between 0-500 nm. In (c) a contour plot is shown for the total light extraction for the investigated NW diameters. The horizontal axis runs from 620 to 640 nm, where the frequency specifies steps of 2 nm within this wavelength range. Two horizontal white dashed lines are used to indicate the light extraction for the NW geometries that show the highest absorption in (b). In (d) the front (forward) light extraction of the structures is included.

[1] Visser, D., Yapparov, R., De Luca, E., Swillo, M., Désières, Y., Marcinkevičius, S. & Anand, S. Top-down fabrication of high quality gallium indium phosphide nanopillar/disk array structures. *Proc. IEEE NMDC* **2019**, (2019).
